# Current Trends in Root Canal Irrigation

**DOI:** 10.7759/cureus.24833

**Published:** 2022-05-08

**Authors:** Azhar Ali, Anuradha Bhosale, Swapnil Pawar, Ateet Kakti, Anjali Bichpuriya, Muhammad A Agwan

**Affiliations:** 1 Endodontics, Ministry of Health, Kingdom of Saudi Arabia, Al Jawf, SAU; 2 Periodontology, Dr D Y Patil Dental College and Hospital , Dr D Y Patil Vidyapeeth, Pune, IND; 3 Endodontics, Yogita Dental College, Khed, IND; 4 Dentistry/Pediatric Dentistry, Riyadh Elm University, Riyadh, SAU; 5 Conservative Dentistry and Endodontics, Bhabha College of Dental Sciences, Bhopal, IND; 6 Restorative Dentistry, College of Dentistry in Alrass, Qassim University, Alrass, SAU

**Keywords:** endodontics, activation, irrigation devices, irrigants, root canal irrigation

## Abstract

Chemical and mechanical root canal debridement are the primary methods used in endodontic therapy to remove all dead tissue, bacteria, and microbial byproducts from the canal. Sodium hypochlorite, a powerful organic tissue dissolver with a broad spectrum of antibacterial properties, is an excellent choice for disinfecting surfaces. Chelating agents, on the other hand, may be used to remove the inorganic components found on the smear layer. This irrigation method is capable of removing the smear layer; however, it is less effective in the apical third. While using irrigant activation devices, irrigating solutions need to be in direct contact with the whole root canal wall surfaces, especially in the apical portions of tiny root canals. The role of irrigants is extremely important because they help not only to clean the canal but also to allow the seepage of the medicaments into the canal system. Thus, the canal needs to be healthy before the obturation procedure. Nowadays, many irrigants have been studied and hence compilation of the various available sources and their effect has to be studied both *in vivo* and *in vitro*. The correlation between the irrigants and the canal cleanliness is of utmost importance, as the success of endodontic root canal treatment depends on its proper activation and characteristics. Hence, this review incorporates the current use of various irrigating solutions and their advantages and disadvantages. In the future, endodontists may employ the novel irrigants and irrigant activation devices that were discovered in this study.

## Introduction and background

Endodontics is a specialty that deals with the anatomy and function of the pulp and periradicular tissues surrounding a tooth's root canals. The goal of root canal therapy is to clean the infected pulp and periradicular tissues while also preventing infection (European Society of Endodontology, 2006). Microorganisms or microflora, which are found in the human mouth, are the most common cause of pulpal and periradicular pathologies [1]. The oral bacterium can form biofilms on the hard and soft tissues of the mouth. Identifying and treating the underlying causes is the main objective of endodontic therapy. Root canal debridement, irrigation, and biofilm removal are all part of the treatment plan for endodontic disease prevention and control [2]. The three main steps of therapy are: root canal preparation, chemo-mechanical debridement, and obturation. Chemical-mechanical debridement requires both instrumentation and irrigation [3]. The goal of the instrumentation is to prepare the canal system for the administration of locally utilized drugs and the placement of a root canal filling [4]. Before using instruments and during the operation, irrigation was used as a pre-instrumentation phase to eliminate contaminated necrotic tissue. Water irrigation has been more important for effective root canal therapy in the last two decades. Root canal instrumentation, irrigation, and medication have been the focus of research and clinical practice, followed by obturation and the placement of a coronal seal. When it comes to water purification, instruments shape and irrigants cleanse. It is impossible to physically disinfect or clean every part of a root canal system. Only an irrigation solution can effectively clean these spaces (main canal, lateral, accessory canal and isthmus) [5].

## Review

History of irrigants

Taft recommended the use of irrigants for root canal irrigation regularly. He suggested using a 'deodorizer' such as sodium chloride. Using a range of flushing chemicals and medicaments, the early literature outlines several procedures for getting a clean canal. To eliminate necrotic pulp tissue, potassium and salt were introduced into the canals. The sulphuric acid solution was put on a cotton pellet and sealed into the root canal for 24-48 h. Callahan was the first to use this method in 1894. After that, a bicarbonate soda solution was injected into the root, causing effervescence and bringing debris to the surface for removal. As a result of research conducted by Grossman and Meiman in the late twentieth century, double-strength sodium hypochlorite was used to remove pulp tissue pieces and dentin shavings after mechanical instrumentation in 1941, which led to the development of the usage of hydrogen peroxide as an irrigant. In 1943, Grossman published a book as a follow-up to his research. Hydrogen peroxide is now being used in clinical practice and has been deemed safe and effective [6].

Function of irrigation

Irrigation is an important part of root canal treatment because it removes dentin shavings from canals. As a result, they do not become compacted near the root canal's apex. Due to the lack of lubrication in dry canals, instruments are unable to work properly. They become more efficient in wet canals. Devices are less likely to break when canal walls are greased by irrigation. They operate as a necrotic tissue solvent, releasing debris, pulp tissue, and germs from uneven dentinal walls when in contact with the substance. They assist in the clearance of debris from auxiliary and lateral channels where instruments are unable to reach. Although they may be antibacterial, the majority of them are germicidal, having a whitening impact on teeth discoloured by trauma or hefty silver restorations. The use of lubricating agents (RC prep, REDTAC, Glyde, etc.) together with irrigants in the canal makes instrumentation simpler and smoother, but the lubricant alone does not make instrumentation easier or smoother [5].

Root canal irrigation solutions

Irrigation solutions should have as few adverse effects as possible so that both the root canal cleansing and biofilm removal are improved. They provide lubrication and a wide antibacterial effect against diverse species established in biofilms, as well as deactivation of bacterial endotoxin in the root canal. They, physically, enable the irrigant to flow through the root canal. A biocompatible, nonirritant and nontoxic irritant should be used [7].

Root canal irrigants have been studied extensively throughout the years, with several research studies examining the effectiveness of different treatments. Although water and normal saline are compatible, instrument debridement alone is inadequate to remove pulp tissue, debris, and bacterial biofilm from the root canal. Antibacterial medications, such as sodium hypochlorite (NaOCl), chlorhexidine gluconate (CHX), EDTA, and a combination of doxycycline, were used as irrigants to test their effectiveness.

Types of irrigation solutions

Normal Saline

In endodontics, normal saline is one of the solutions used as an irrigant. It results in root canal debridement and lubrication. Because of its moderate activity, it may be used in conjunction with chemical irrigants. After root canal preparation, it may be used as a last rinse to flush out any leftover chemical irrigant. The most common saline solution is 0.9 percent W/V normal saline [5].

Sodium Hypochlorite

Sodium hypochlorite (NaOCl) liquid, which is clear and light greenish-yellow, has a predominating chlorine smell. Upon exposure to light, it degrades and becomes water-soluble [8]. Surveys from across the globe show that sodium hypochlorite is the most often utilized irrigating solution in endodontics.

Sodium hypochlorite has several advantages. It is an antibacterial and proteolytic agent that works as a fantastic organic tissue solvent. It is a lubricant with a quick onset of action. It is widely used both as an oxidizing and a hydrolysing agent. Unfortunately, despite its many positive characteristics, NaOCl has significant drawbacks as well, including the fact that it is poisonous, ineffective in removing smear layers, corrosive, it may result in discolouration, and pungent odour. The sealer's connection to the dentin will be compromised if NaOCl is used as a final rinse. The percentage of NaOCl to be used during the entire cleaning and shaping treatment is recommended to be between 2.5 and 6%.

Mechanism of Action of Sodium Hypochlorite

Hypochlorite (OCl^-^) and hypochlorous acid (HCl^-^) are the two most reactive forms of chlorine in an aqueous solution at body temperature (HOCl). In an aqueous solution, when the pH of water is less than 7.6, hypochlorite is prevalent, whereas hypochlorous acid is prevalent when the pH value exceeds 7.6. Because sodium hypochlorite contains 5% free chlorine, proteins are broken down into amino groups. Sodium hypochlorite, a commonly used disinfectant, releases its OCl form at a pH of 12. Hypochlorite dissolves necrotic tissue because of its strong alkaline nature (pH 12). A buffering agent, 1% sodium bicarbonate, is added to increase the effectiveness of the NaOCl solution. However, buffering reduces the shelf life of the solution to less than a week [5].

NaOCl activity is influenced by the following factors.

*Concentration.* When the concentration of the sodium hypochlorite solution is reduced,​​​​ its toxicity, antibacterial action and ability to dissolve tissues are all reduced. When the volume of an irrigant is increased, the chances of drastically reducing bacteria colonies in the root canal are more.

*Time. *To maximize its antibacterial properties, sodium hypochlorite must remain in contact with the canal for a longer duration of time than it does for shorter periods. In necrotic cases, this is very important. To boost the activity of a low-concentration NaOCl solution, increase its temperature. NaOCl at 45°C has the same tissue-dissolving ability as a 5.25% solution at 20°C, according to the results of this study. Furthermore, heated low-density NaOCl solutions have lower systemic toxicity than unheated, higher-concentration NaOCl solutions.

Chlorhexidine

Chlorhexidine (CHX) is a strong antiseptic that is often used to chemically control plaque in the mouth. Mouthwash is made up of 0.1-0.2% aqueous solutions, while root canal irrigation in endodontic treatment is done with a 2% concentration. The antibacterial action of CHX is dependent on achieving an ideal pH (5.5-7). At lower quantities, CHX is bacteriostatic; at larger quantities, it is bactericidal. CHX is effective against Gram-positive and -negative bacteria, spores of bacteria, lipophilic viruses, yeast, and fungi. But since CHX is pH-dependent, these effects are much reduced when organic matter is present. Aside from destroying bacteria, CHX is incapable of removing biofilms and other organic debris. A CHX solution of 2% after the chemo-mechanical preparation provides the appropriate antibacterial effect. Calcium hydroxide (Ca(OH)_2_) is a common intracanal medication in this solution. One of the reasons for the extensive use of CHX is it attaches to hard tissues and retains its antibacterial action. This is because of the interaction of a large number of CHX molecules with dentin at any point in time. According to White et al., 2% of CHX produced effects lasting from 72 hours to 12 weeks. The main drawback of CHX is its inability to dissolve in tissue. CHX is a matrix metalloproteinase (MMP) inhibitor with a wide range of action (anti-collagenolytic effect) [9].

For a stronger connection, add CHX to the dentinal tubules by applying it directly to the surface of the dentin. Size and structure have a direct correlation to the toxicity of CHX. However, although CHX does not cause long-term harm to host tissues, if it is mistakenly extruded from root canals or injected, it might provoke an inflammation reaction. Dental and oral pigmentation, desquamative gingivitis and a poor taste in the mouth are just a few unusual side effects that CHX may cause in some people (bad metallic taste in the mouth). Antibacterial effectiveness is increased by heating the CHX solution at a low concentration, while toxicity is reduced. CHX may be used to disinfect gutta-percha. Increased action against bacteria and biofilms occurs when surface-active compounds are added to a CHX product (CHX-Plus) that has lower surface tension. The possible complications that arise when a surfactant-containing irrigation fluid spills from the periapical tissues in clinical practice have not been studied yet. To complete the canal cleaning process, QMix is a root canal irrigation product. CHX is mixed with a surfactant and ethylenediaminetetraacetic acid (EDTA) to better penetrate the dentinal tubules.

EDTA

Tissue-dissolving irrigation solutions, both organic and inorganic, are essential for a comprehensive root canal cleanup. To remove the smear layer or other debris from the root canal system, NaOCl, which dissolves only organic tissue, should not be used. A supplementary solution of EDTA and other demineralizing agents should be administered during root canal therapy. In 1957, Nygaart-Ostby recommended the use of chlorinating chemicals for the production of hardened root canals. In the beginning, it was advised to use the 15% EDTA solution with a pH value of 7.3. The most common kind of EDTA solution is a neutralized solution with a concentration of 17%. Dentin calcium ions react with fluid to generate calcium chelates. When the chelator is missing, the process of decalcification halts. Calt and Serper used 10 mL of the EDTA solution for 1 min to remove the smear layer of the canal wall. Demineralization of dentin was shown to increase with the amount of time spent in contact with it. In the apical third of the root, a minute-long ultrasonic application of 17% EDTA is highly helpful, and the use of liquid EDTA during root canal therapy is also advised [10].

Citric Acid

The citric acid (CA) is available on the market in quantities ranging from 1% to 50%. Using 10% CA as a final irrigation solution offered good results for removing smear layers. Although EDTA and CA are equally effective in removing the smear layer from root canal walls, CA has shown some advantages over EDTA when used at comparable doses. The cytotoxicity of chelators has been studied *in vitro*. The biocompatibility of a 10% CA solution against a 17% EDTA solution has been shown. On three separate occasions (1, 5, and 10 min), a 25% CA solution was shown to be ineffective in the removal of *Enterococcus faecalis* biofilms [11].

Mixture of Tetracycline Isomer, Acid, and Detergent

Torabinejad et al. used Tween-80 instead of EDTA to enhance the removal of the smear layer [12]. This mixture (mixture of tetracycline isomer, acid, and detergent - MTAD) has antibacterial and chelating characteristics. Because it does not dissolve organic tissues, it is best to use this after NaOCl at the end of the chemomechanical preparation step. MTAD is made up of three different compounds that together are expected to have a potent antibacterial action. *E. faecalis* biofilm is more susceptible to NaOCl solution's bactericidal action at concentrations of 1-6%. The smear layer removal with CA is conceivable, allowing the antibacterial effects of doxycycline to penetrate the dentinal tubules. The bond strength of MTAD is much lower than that of the final irrigation solution of EDTA in canals with AH Plus and gutta-percha. Root canal bacteria may become resistant to tetracycline if MTAD is used in place of EDTA. A biocide such as NaOCl or CHX is usually preferred over an antibiotic since antibiotics were designed for systemic rather than local use and have a restricted scope [12].

Tetraclean

Tetraclean has a process similar to that of MTAD, but with a lesser dose of doxycycline and detergent. Propylene glycol is the detergent type, whereas the antibiotic concentration is 50 mg/ml of doxycycline, which differs from what is utilized in MTAD. Because it will not dissolve organic tissue as NaOCl does, tetraclean is best used at the end of the chemomechanical preparation after NaOCl [13]. This product has a lot of power against both facultative and anaerobic bacteria. Planktonic and *in vitro* biofilm *E. faecalis* cultures as well as mixed-species biofilms respond better to tetraclean than MTAD [13].

Etidronic acid or etidronate (HEBP), a decalcifying substance, interacts very little with sodium hypochlorite (NaOCl). A substitute for EDTA or CA has been proposed. In the treatment of osteoporosis and Paget's disease, HEBP is a systemic drug that decreases bone resorption. To determine whether this therapy shortens or lengthens endodontic irrigation, an additional study is required. It takes longer to remove minerals from the body with 9% or 18% HEBP than it does with 17% of the same concentration of EDTA.

Superoxide Water

Superoxidized water is obtained by the electrochemical treatment of a saline solution. It can be obtained from regular tap water and low-concentration salt solutions by electrochemically activating (ECA) them.

Oxidizing compounds having microbicidal action against bacteria, viruses, fungi, and protozoa make up anolyte solutions. The names 'superoxidized water' and 'oxidative potential water' are both used to describe this kind of water. They are non-toxic and do not harm key biological tissues [14]. ECA has promising results for effective root canal irrigation.

Ozonated Water

Even at low quantities, ozone (O_3_) can kill pathogens, including spores (0.01 ppm). It is simply prepared using an ozone generator. Ozone dissolves quickly and easily in water. Lipopolysaccharides in root canals were discovered to have biological consequences, including the induction of apical periodontitis and could not be neutralized by ozonated water, despite the fact that ozonated water kills bacteria. Before ozonated water is used as a frequent therapeutic technique for root canals, more research is needed [15].

Factors influencing intracanal irrigant activity

The tissue dissolving power of NaOCl is higher at 5.2% than at 2.5% and 0.5%, and therefore, the higher the concentration, the greater the effectiveness [5].

Touch: To be effective, the irrigant must contact the substrate. The presence of organic tissue must be removed for irrigation to be successful.

Quantity of irrigant utilized: The more irrigant is used, the more effective it is. Irrigating needle gauze: 27 or 28 gauze is used for improved canal penetration.

Irrigant's surface tension: The lower the surface tension, the better the wettability.

Irrigant's temperature: Warming the NaOCl boosts its efficacy.

Irrigation frequency: The higher the frequency, the better the outcomes.

Canal diameter: The wider the canal, the better the irrigant's effect.

Irrigant's age: Newly produced solutions are more efficient than older solutions.

Irrigation techniques

Irrigation's effectiveness and safety are determined by the method of supply. An open-ended needle on the needle of the classic irrigation device enables the irrigant solution to enter and flush the canals of debris. While irrigating the canal, keep the following in mind: (1) The solution must be gently and passively inserted into the canal. (2) The needle should never be forced into the canal, and there should be plenty of room for backflow during the injection. (3) Use a needle with a 25 or 27 gauge. (4) Using the pulp chamber if the canals are tiny is preferable. After that, a file will be used to transport the solution into the canal. (5) Irrigation is dependent on the canal's size and shape. If the apical region is to be adequately disinfected, the canals must be stretched to a diameter of at least 30. (6) It is imperative that irrigants are not pushed into the tissue, but rather gently inserted into the canal, regardless of how they are delivered. (7) It is critical that the needle delivering the cleaning solution be placed close to the object being cleaned. Insert the needle tip into the canal until resistance is felt and then withdraw the needle and irrigate the canal passively. (8) To effectively clean both front and posterior dental canals, a 30-degree blunt bend in the needle's core may be used. (9) Irrigation solution volume is more important than irrigant concentration or sort [16].

Size and patterns of needle tips

Although 25-gauge needles were formerly the norm for endodontic irrigation, 27-, 30-, and even 31-gauge needles have subsequently taken their place.

27 G and 30 G are also identical to ISO sizes 0.42 and 0.31, respectively, hence smaller needles are recommended. Root canal irrigants can only penetrate so far because of the dead-water zone or sometimes air bubbles that prevent the solution from reaching its full potential. A potential drawback of this technique is that the irrigant is given too far from the apex because of the small needles. Following changes to the needle design have been made to ensure that the desirable attributes of needles (blandness) are met: let the water flow backwards; flexible; the length is longer; it is easily accessible; and budget-friendly.

Syringes

The most common irrigation equipment is a plastic syringe with a size range of 1-20 mL. Even though they cannot control pressure and are error-prone, large-volume syringes may be preferable for speed. The 1-5-mL-sized syringes are the best choice for safety and control rather than the bigger ones. Luer-Lok endodontic irrigation syringes are required. Using separate syringes for each solution is preferable because of the chemical interactions between various irrigants.

Brushes

To put it another way, canals are not irrigated directly with brushes. Adding them to the procedure will aid in cleaning out the canals and stirring up the root canal solution. Through an indirect method, they may be involved in the transportation of irrigants throughout canal areas. For the first time, commercially available irrigation needles wrapped in a brush-like substance have been made available (NaviTip FX, Ultradent Products Inc., South Jordan, UT). A recent study found that the coronal third of instrumented root canal walls was found to be cleaner using the NaviTip FX needle than the brushless sort of the NaviTip needle. In certain cases, radiolucent bristles may be displaced by friction between the bristles and canal abnormalities, even using a surgical microscope. In the early 1990s, Keir et al. revealed that the use of canal brushes increased canal debridement. They furiously brushed and turned the Endobrush. Twisted wires hold nylon bristles, which are attached to a handle of the same diameter as the brush's length, which is manufactured by C&S Microinstruments Ltd, Markham, Ontario, Canada. Because the Endobrush is so tiny, it may cause debris to accumulate at the canal's apex after cleaning.

Agitation

Removing debris and germs from the root canal system in a more effective way has long been a goal of root canal irrigants. If the irrigant is agitated and dispersed throughout the root canal system, it may better achieve its physical and chemical goals by creating shearing and streaming pressures [17]. Two types of agitation procedures are available: manual and rotational. With needles, brushes and manual dynamic agitation with files or gutta-percha points, manual irrigation methods are all instances of this kind of treatment. Rotary irrigation methods include rotating brushes, continuous irrigation during instrumentation, sonic and ultrasonic vibrations, and the application of negative pressure during root canal irrigation. When compared to traditional syringe needle irrigation, these methods result in cleaner canals.

Manual Agitation Techniques

Before passive ultrasonic activation, traditional irrigation with syringes was considered a successful way of irrigant delivery. In this method, an irrigant is introduced into a dental canal either passively or by moving the needles/cannulas up and down in the canal space. As a result, the apex and canal walls of the irrigation system as well as its flow pattern, velocity and depth of penetration may be affected significantly. Irrigants can only go as deep as their irrigation tip gauge allows them to. A 21-gauge tip is required to reach the apex if you have an ISO 80 canal. A 23-gauge tip is required if the canal is 50 gauge. A 27-gauge needle is often used in endodontic operations. Many studies showed that the irrigant has only a limited effect beyond the needle tip due to the apical root canal's deadwater zone or sometimes air bubbles. There are both manual and dynamic control over needle penetration and the quantity of irritant released from the canal. Canal walls must be in direct contact with an irrigant in order for it to work. Due to the vapour lock effect, it is difficult for the irritant to reach the canal's apical portion. For example, this kind of irrigation has been shown to have a significant hydrodynamic influence and significantly increase the exchange of chemicals in an instrumented canal. This assertion has been supported by a recent research study conducted by McGill et al. and Huang et al. Studies conducted by Duerr Dental Co. in Bietigheim-Bissingen, Germany, found that manual-dynamic irrigation (RinsEndo) was much superior to static irrigation and an automated dynamic irrigation system (RinsEndo) [18].

Manual-Dynamic Irrigation

The manual-dynamic irrigation technique has many advantages: (1) Irrigation is delivered to untouched canal surfaces more effectively due to the push-pull action of a well-fitting gutta-percha point in an open canal. (2) The frequency of the push-pull motion of the gutta-percha point (3.3 Hz, 100 strokes per 30 s) is higher than the frequency of the positive-negative hydrodyne motion (1.6 Hz). With the second method, the fresh, unreacted solution is mixed well with the used, reacted irrigant. Because it is so labour-intensive to do by hand, manual-dynamic irrigation has not found widespread use in clinical settings despite its promotion as a simple and inexpensive canal irrigation method. Automated root canal irrigation systems are either commercially available or being developed by manufacturers as a consequence [18].

Technique of mechanical agitation

Rotary Brush

This includes both Ruddle brush and Canal brush.

The instrumented root canals were cleaned with a microbrush fitted to a spinning handpiece. The brush features a shaft and a tapered brush section. From a central wire core, many bristles protrude out in all directions in a second. At roughly 300 rpm during debridement, the microbrush's bristles are forced to bend into the flaws of the preparation. As a result, the canal may be cleared of any residual material in a coronal direction. Although this gadget was patented in 2001, it has not yet been commercialized. The Canal brush is another endodontic microbrush just made commercially available. This microbrush, which is constructed entirely of polypropylene, can be rotated and used as a manual tool. Townsend amd Maki found that debris from simulated canal extensions and flaws was efficiently cleaned using a compact and flexible Canal brush and an irrigant [19].

During rotating irrigation, water is applied continuously. An independent fluid supply device is used when employing the Quantec-E Endo System in conjunction with the Quantec-E Irrigation System. A pump console, two irrigation reservoirs and tubing are used to keep the instruments irrigated at all times while they are rotating.

Sonic waves may also be used for irrigation. They were the first to describe the use of sonic devices in endodontic procedures. Ultrasonic irrigation generates more shear stress and has a higher frequency (1-6 kHz). Sonic irrigation has lower shear stress and a lower frequency. Tronstad et al. [20] found that sonic irrigation using noncutting polymer tips is known as the Endo Activator and is used for therapy. A research study found that this method is proved to be effective.

Battery-powered vibrations (9000 cpm) are combined with manual root canal irrigation in the Vibringe system. Using sound waves to provide medicine is one of the Vibringe's distinctive characteristics [21].

Ultrasonically or subsonically controlled ultrasonic or subsonic handpieces are two types of irrigation tools. Ultrasonic handpieces, which feed sound waves into the endodontic file, cause the file to vibrate at a rate of 25,000 times per second. There is an audible flow of water, as the irrigant cuts through the dentin. A further effect of cavitation in the irrigating fluid is the movement of debris. There are two types of ultrasonic irrigation: the first one uses irrigation in conjunction with ultrasonic devices (UI). The second is passive ultrasonic irrigation (PUI) as it requires no additional equipment. PUI eliminates more pulpal tissue and dentine debris than syringe needle irrigation because of the increased irrigating flow and velocity in the canal. Ultrasonics are worthless if the apical vapour lock prevents them from cleaning the root canal system of dirt and bacteria. According to Ruddle, at the apex, positive and negative pressures exist. It seems that there are two contradictory occurrences when it comes to utilizing syringe needles to give irrigants: 'Debridement and smear layer clearance of debris require that the irrigants be in close contact with the canal walls during irrigation. When the needle tips are too far apart, it becomes difficult for the irrigants to reach the apical portions of the canal'. There is a larger danger of irrigant extrusion when the needle tips are too near to the anastomosis foramen, which might cause considerable iatrogenic harm to the tissues around the foramen. The use of pressure alternation devices to give irrigant and aspirate simultaneously provides a reasonable solution to this challenge.

Devices that Change the Pressure

RinsEndo and EndoVac are two examples of negative-pressure irrigation systems. To irrigate the canal, RinsEndo uses pressure suction technology. It consists of a handpiece, a 7-mm cannula and an irrigant syringe [22]. The EndoVac, an apical negative pressure irrigation system, consists of a master delivery tip (MDT), a macrocannula, and a microcannula. The MDT irrigates the pulp chamber while also removing the irrigant from it. A dental unit consists of a high-speed suction and an irrigant syringe where both are connected to the microcannula and microcannula through tubing on the devices. An irrigant is sucked up to the canal's middle part using the macrocannula, a polypropylene flexible tube having an open end diameter of 0.55 mm, an internal diameter of 0.35 mm and a taper of 0.02 mm. The microcannula is made of stainless steel and includes a total of 12 tiny holes placed in four rows of three at its apical 1 mm. The first hole in the row is 0.37 mm from the microcannula's tip, and there is a 0.2-mm gap between each of the other holes in the row. Aspirating irrigants and debris from canals with a diameter of 35 or more requires using a microcannula with an outer diameter of 0.32 mm. The working length (WL) of the microcannula should be used for this purpose. Water from the irrigation system is sent via the MDT, which removes any excess water from the pulp chamber before it may overflow. By creating a negative pressure in the cannula, the MDT draws the irrigant from its fresh supply inside the chamber through the canal and out of the cannula through the suction hose. To keep the WL constantly irrigated, a negative pressure is used. The EndoVac and needle irrigation methods were investigated by Nielsen et al. [23] for debriding the apical 3 mm of a root canal. There was no difference between the two irrigation systems at the apical 3-mm level. The EndoVac technology, on the other hand, resulted in much less debris at the 1-mm apical level. Microbial management was also demonstrated to be better with the Endovac irrigation system than with the typical irrigation delivery system. EndoVac, according to another *in vitro* investigation, left substantially less material behind than the traditional 30-gauge needle irrigation procedures.

## Conclusions

The removal of bacteria during cleaning and shaping is critical to endodontic success. The fact that the irrigant must be utilized in such a manner that it may function to its full capacity in the root canals should always be bear in mind. Different practitioners use different irrigants. Irrigants that totally eradicate germs and clean the root canal have yet to be discovered. Although NaOCl has a few downsides, it is the most often used irrigant in daily clinical practice. The correct application of the necessary irrigant aids in achieving a sufficient antibacterial action and so improves endodontic success.
